# New-onset refractory status epilepticus after SARS-CoV-2 infection: a review of literature

**DOI:** 10.3325/cmj.2024.65.510

**Published:** 2024-12

**Authors:** Athanasios Stavropoulos, Dimitrios Filippou

**Affiliations:** 1 Medical School of Athens, National and Kapodistrian University of Athens, Athens, Greece; 2 Research and Education Institute in Biomedical Sciences, Pireaus, Greece

## Abstract

Although severe acute respiratory syndrome coronavirus 2 (SARS-CoV-2) primarily affects the respiratory system, neurological symptoms were reported both during acute and post-acute COVID-19. Notably, patients with no history of epilepsy or other neurological conditions developed new-onset refractory status epilepticus (NORSE) weeks, months, or even up to a year following the viral infection. While NORSE is uncommon, it carries a high mortality rate and can result in permanent epilepsy. Therefore, clinicians should consider the possibility of death or epilepsy development when treating individuals with NORSE who have recently contracted SARS-CoV-2. This article compiles comprehensive information on the mechanisms of epileptogenesis linked to SARS-CoV-2, the diagnosis of NORSE syndrome, its treatment options, and associated outcomes. Our aim was to enhance physicians' understanding of the virus's pathogenesis and increase the awareness of NORSE.

Severe acute respiratory syndrome coronavirus 2 (SARS-CoV-2) emerged in late 2019, and the COVID-19 pandemic was officially declared on March 11, 2020 (1). While primarily affecting the respiratory system, SARS-CoV-2 is also associated with a range of symptoms that can linger for months following the initial infection. These post-COVID symptoms can affect the respiratory, cardiovascular, or nervous systems (2,3). A notable neurological complication arising from COVID-19 is new-onset refractory status epilepticus (NORSE). Over the past few years, numerous cases of NORSE have been linked to SARS-CoV-2 (4). Researchers believe that the virus infiltrates the central nervous system (CNS) and engages with cortical neurons, leading to the emergence of NORSE several weeks, months, or even up to a year following the viral infection (5,6). Seizures may also arise in the context of post-COVID complications, including stroke, encephalitis, and multisystem inflammatory syndrome (MIS) (5-10). It is crucial for researchers to recognize the possibility of post-COVID NORSE, given its significant mortality risk and the potential to cause epilepsy.

NORSE occurs in previously healthy individuals without a history of epilepsy or other neurological issues. Status epilepticus (SE) is characterized by prolonged seizure activity or the occurrence of two or more seizures within 5 minutes. When SE persists despite the administration of two standard intravenous antiepileptic drugs, one of which must be a benzodiazepine, it is referred to as refractory status epilepticus (RSE) (5,6,11-16). NORSE indicates RSE with no clear underlying cause. However, the majority of NORSE causes are typically determined within 24-72 hours (17,18). These causes are commonly linked to strokes, head injuries, or infections affecting the CNS. Infections can be caused by bacterial pathogens such as syphilis, tuberculosis, or bacterial meningitis; viral infections like varicella-zoster virus (VZV) or HIV; or even fungal organisms (19-22). The incidence of NORSE is approximately 12.6 per 100 000 in the US, Europe, and Asia, with the mortality rate ranging from 16 to 27% (10).

There are many literature reviews addressing NORSE cases after SARS-CoV-2 infection. The novelty of our study, aimed to increase the awareness of NORSE, is the synthesis of data regarding the pathogenetic mechanism, diagnosis, treatment, and possible outcomes of post-COVID NORSE. 

## Methods

**Figure 1:**
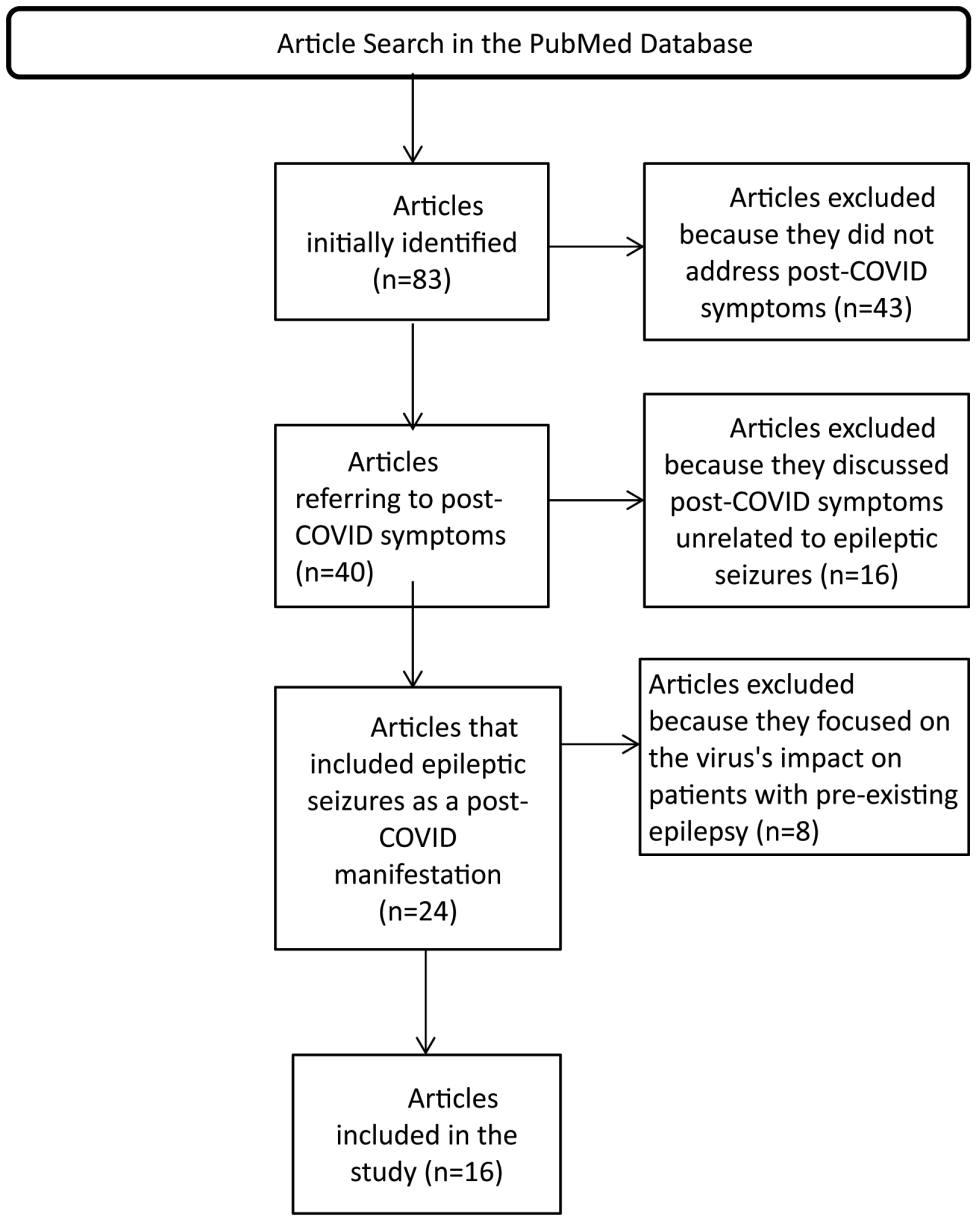
The article selection process.

PubMed was searched in November 2023 using the following keywords: “post-COVID epilepsy,” “status epilepticus,” “epileptogenesis,” and “nervous system.” In total, 83 articles were initially identified. Of these, 43 were excluded because they did not address post-COVID symptoms, 16 because they discussed post-COVID symptoms unrelated to epileptic seizures, and 8 because they focused on the virus's impact on patients with pre-existing epilepsy. Ultimately, from the original 83 articles, 16 were included in the study (Figure 1). Only English-language publications were considered, encompassing case reports, narrative reviews, and systematic reviews, while studies involving animals were not included.

## Results

All 16 articles (5,7,9,22-34) addressed patients who developed NORSE in the context of SARS-CoV-2 infection and the frequency of their epileptic seizures until they were treated according to the epilepsy protocol. Of these, 6 attempted to explain the mechanism of the virus entry into the CNS (5,9,22-24,27) and 3 addressed its molecular interaction with neurons (9,22,24). Furthermore, 8 articles investigated NORSE manifestation following post-COVID complications: stroke (9,22), encephalitis (5,9,22,23), and MIS (7,9,22,25-27). Lastly, 12 articles (5,7,22-28,30,32) presented the treatment protocol for NORSE and 11 (5,7,22-28,31,32) focused on factors associated with the onset of epilepsy in these patients. Only 3 articles (22,24,28) referred to a small number of patients who developed epilepsy over the course of a few months.

## Discussion

### Mechanism of CNS entry

There are two theories regarding the neuroinvasion of SARS-CoV-2: the neuronal retrograde trans-synaptic pathway and the vascular pathway (9,10,35-37).

The initial phase of the neuronal trans-synaptic pathway involves the virus directly attaching to the ACE2 receptors present on olfactory sensory neurons (OSN). These neurons reside in the olfactory epithelium and are bipolar in structure. Their dendrites, which act as binding sites for the virus, are situated in the olfactory mucosa of the nasal cavity, while their nerve terminals project to the olfactory bulb (35,38). Once bound, the virus is encapsulated in small vesicles and transported along the axons of the cells that traverse the cribriform plate. It is released from the OSN through exocytosis and passed on to the olfactory neurons in the olfactory bulb, a process known as trans-synaptic transmission. After this, vesicles containing the virus travel down the axons of the olfactory neurons, spreading to various regions of the brain (35,38-40).

The vascular pathway begins when the virus attaches to ACE2 receptors present on the epithelial cells lining the nasal passages, airways, and intestines. This invasion of host cells by the virus facilitates its replication and the release of viral particles into the systemic circulation (35,41). The release may incite a systemic inflammatory immune reaction, which could compromise the integrity of the blood-brain barrier (BBB). This can occur directly through the release of pro-inflammatory cytokines, or indirectly via bradykinin, which interacts with bradykinin-2 receptors on endothelial cells (24,35). BBB disruption allows the entry of viral particles into the CNS (7,9,25-27,35,36,42-46). When vascular inflammation escalates into a prolonged systemic inflammatory response triggered by an overproduction of cytokines, it is referred to as MIS (7,8), which will be explored further below.

However, the virus is speculated to be able to enter the CNS without disrupting the BBB by infecting macrophages and leukocytes, which enter the CNS freely. This mechanism is referred to as the “Trojan horse” mechanism (34,47,48).

### Cellular and molecular mechanism of epileptogenesis associated with COVID-19

Following the infiltration of inflammatory cells and viral particles into the CNS, a range of substances is released that activate cortical neurons. These substances enhance the function of excitatory glutamate receptors, including *N*-methyl D-aspartate (NMDA) and non-NMDA receptors (such as AMPA and kainate receptors), while simultaneously reducing the activity of inhibitory GABA-A receptors (35,49-57). Specifically, inflammatory cells release nitric oxide, prostaglandin E2, and various inflammatory cytokines, such as tumor necrosis factor-α, interleukin-1, and interleukin-6 (9). Additionally, neurons affected by the virus produce SARS-CoV-2 proteins, which resemble the body's neurotransmitters. These viral proteins exert effects akin to those of excitatory neurotransmitters glutamate and dopamine, as well as of inhibitory neurotransmitters serotonin and acetylcholine (35,58). Finally, the virus's entry into microglial cells triggers their activation, which results in the release of excitatory inflammatory cytokines and the phagocytosis of neurons, ultimately causing neuronal loss. A reduction in the gray matter has been noted in the limbic, paralimbic, orbitofrontal, and anterior cingulate cortex, with the hippocampus and parahippocampus gyrus in the limbic cortex and the frontal lobes showing the most significant anatomical alterations (42,45). MRI scans in NORSE survivors confirmed the atrophy of the hippocampus and frontal lobes, alongside heightened neuronal damage markers. These markers include S100 β protein, enolase-specific protein, and glial fibrillary acidic protein (35,43). Notably, epileptic seizures exacerbate the activation of microglial cells, creating a feedback loop that further amplifies this activation (43).

NORSE can manifest following post-COVID complications: stroke, encephalitis, and MIS (5-10). Stroke is attributed to endothelial cell inflammation. The virus attaches to ACE2 receptors on endothelial cells, triggering an inflammatory response that activates both intrinsic and extrinsic coagulation pathways. This process elevates fibrin levels in the bloodstream, resulting in thrombosis and ischemic strokes. Hypoxia resulting from an ischemic stroke enhances the release of glutamate, an excitatory neurotransmitter (35,59). An ischemic stroke has the potential to turn into a hemorrhagic event, further increasing hemosiderin deposits in the neighboring neurons. Elevated hemosiderin levels boost free radical production within the neurons, contributing to inflammation and the release of excitatory inflammatory cytokines (9).

Encephalitis, unlike stroke occurring post-COVID, arises after the virus infiltrates the CNS. Neuroinvasion triggers neuronal demyelination, local inflammation, and widespread edema (5,9). These elements contribute to the activation of the surrounding neurons in the edema region, presenting clinically as drowsiness, disorientation, and seizures (5).

MIS is a systemic inflammation that persists even when SARS-CoV-2 is no longer active. To date, SARS-CoV-2 remains the sole known trigger of MIS, which typically manifests 2-6 weeks after the initial infection. The condition primarily affects children aged 5 to 11, but adults have also been affected (28,60,61). As previously noted, MIS develops when SARS-CoV-2 viral particles enter the systemic circulation, leading to prolonged inflammation that can disrupt the BBB, allowing the virus access to the CNS. While the exact cause of MIS is still unknown, several hypotheses have emerged, including persistent infection linked to insufficient antibody responses or an abnormal hyperreactivity of the immune system (7). MIS can present with a range of symptoms: fever, skin redness, conjunctivitis, diarrhea, and vomiting. It can cause dysfunction in one or more organs, including the heart, liver, and kidneys (28,60,61). Diagnosing MIS relies on increased serum inflammatory markers, often referred to as a “cytokine storm,” the presence of anti-COVID IgG in serum, and elevated proteins in the cerebrospinal fluid (CSF) (7,29,43,44,62).

### Treatment options in managing NORSE

Patients diagnosed with NORSE syndrome should be managed according to established epilepsy protocols, which suggest initiating treatment with anti-seizure medications (ASMs) immediately after the first seizure (30,63). Typically, a regimen involving three ASMs is recommended while seizures persist, with patients often requiring hospitalization during this phase. Once seizures have resolved, patients are discharged on a maintenance plan of two ASMs for an extended period. Noteworthy ASMs include levetiracetam, zonisamide, oxcarbazepine, clonazepam, lorazepam, lacosamide, and phenytoin (31,64). The appropriate dosage and combination of these medications should be determined based on EEG and MRI screening results. Patients experiencing adverse effects from first-line ASMs should be offered alternative combinations (45).

The treatment protocol varies slightly for patients diagnosed with MIS. These patients are recommended to take their three ASMs in conjunction with intravenous immunoglobulins (IVIG) and pulse-dose steroids. Administered over five days, IVIGs and steroids are dosed as determined by the physician (7,32,43,62,65). The initial three first-line ASMs continue until seizures are controlled, followed by two ASMs after discharge. Noteworthy, patients with NORSE and MIS who only received ASMs without IVIGs and pulse dose steroids often experienced persistent seizures (5,43,44).

The consequences of NORSE differ significantly: patients may experience a single episode, develop epilepsy, or die. The mortality rates range from 16% to 27%. Patients who survive NORSE relatively rarely develop epilepsy. The majority of patients positively respond to ASMs, IVIGs, and high-dose steroids, which have been demonstrated to lower the likelihood of epilepsy (32,33,65,66). Consequently, seizures either resolve completely or become less frequent. Nonetheless, some patients continue to have seizures one or two years after the initial seizure onset (34,67,68). These patients ultimately develop epilepsy, despite adhering to the prescribed anti-seizure medications.

Several elements are associated with the onset of epilepsy in patients with NORSE syndrome. One significant factor is MIS. “Cytokine storm,” combined with elevated levels of complement components and high mobility group box-1 proteins, persists in the body for an extended period of time. Throughout this period, ongoing neuronal loss and heightened neuronal excitability increase the likelihood of developing epilepsy (35). Additionally, individuals with structural alterations in the hippocampus and frontal lobes face greater risks. In most patients who developed epilepsy, MRI and brain tissue biopsy showed atrophy of these anatomical regions (42,45). Moreover, greater risk of developing epilepsy is present in individuals who experience a stroke linked to SARS-CoV-2. This might be explained by an excessive release of glutamate during an ischemic stroke, which may over time lead to prolonged neuronal stimulation. Likewise, oxidative stress and inflammatory cytokines present during a hemorrhagic stroke might persist for an extended period, lastingly harming the adjacent neuronal cells (35). Additionally, individuals with genetic variations identified in the epilepsy panel may be susceptible to epilepsy, as these variants become activated during NORSE (44).

In the current article, we summarized all the currently available data regarding post-COVID CNS evolvement and the potential mechanisms implicated in the development of post-COVID NORSE, a rare but potentially fatal post-infectious neurological adverse event. We identified and synthesized all the available existing data from studies on PubMed, 14 of which involved hospitalized patients, while 2 (22,30) were review articles. Currently, the exact mechanisms implicating viral pathogenesis and CNS involvement remain elusive, which highlights the need for further research (35,68,69). *In vitro* studies of the activity of the virus are needed in order to understand the pathogenetic process behind the entry into the CNS and the molecular mechanism of epileptogenesis, in combination with the clinical presentation and the patient’s imaging findings.

Importantly, NORSE can arise from infections beyond SARS-CoV-2: bacterial, viral, or fungal. Bacteria such as *Treponema pallidum*, *Mycobacterium tuberculosis*, *Streptococcus pneumoniae,* or *Neisseria meningitides*, which can cause meningitis, are able to infiltrate the CNS and induce epileptic seizures. Additionally, viruses that may provoke status epilepticus include herpes simplex virus type 1, VZV, Epstein-Barr virus, cytomegalovirus, HIV, and arboviruses. Fungal pathogens may also be involved, whether they are yeast forms like *Cryptococcus spp*, molds such as *Aspergillus spp*, or dimorphic fungi exemplified by *Histoplasma capsulatum* (21). All of these infections can contribute to NORSE, with or without subsequent post-infectious complications. Such events encompass encephalitis and strokes, frequently triggered by infectious agents. Therefore, physicians need to distinguish between non-infectious and post-infectious encephalitis or strokes that lead to NORSE, as this distinction will affect the treatment. Additionally, NORSE may arise after significant head trauma, which requires physicians to promptly rule out other potential causes (19-22).

In the context of post-COVID NORSE, physicians need to determine whether the event is associated with MIS. MIS is difficult to diagnose, as patients might be asymptomatic or exhibit only a subset of diverse symptoms typical of the syndrome (28,60,61). Hence, physicians should test for elevated serum inflammatory markers and anti-COVID IgG, and increased CSF proteins. The exact diagnosis is paramount as NORSE associated with MIS requires different treatment. Furthermore, an efficient target therapy must be developed in order to minimize the risk of failure of the current treatment (32,65).

After treating NORSE, health care providers must take all the necessary steps to mitigate the risk of developing epilepsy (34,67-69). Specifically, they should screen for MIS and, if necessary, initiate the appropriate treatment. Furthermore, MRI scans and brain tissue biopsies should be used to identify any atrophy in the hippocampus or frontal lobe. Similarly, post-COVID stroke should be ruled out. Lastly, genetic testing can serve as a valuable resource to identify any genetic variants from the epilepsy panel that patients may be unaware of.

### Conclusions

While post-COVID NORSE is an uncommon adverse event associated with SARS-CoV-2, its severity and possible fatal consequences underscore the necessity for additional research. Notably, NORSE can manifest weeks, months, or even a year following the initial viral infection. Healthcare providers must approach each patient carefully, as the appropriate treatment may vary depending on the specific pathogenesis involved. Ultimately, clinicians should always consider the risk of developing epilepsy, which reinforces the importance of a comprehensive patient evaluation.
